# Vitamin D in pediatric rheumatology

**DOI:** 10.1186/1546-0096-12-S1-I28

**Published:** 2014-09-17

**Authors:** Jelena Vojinovic

**Affiliations:** 1 Department for Pediatric Rheumatology, Clinical Center, Faculty of Medicine University od Nis, Nis, Serbia

## 

One century after its discovery vitamin D is shown to be, in fact, a pleiotropic steroid hormone which, beside regulation of calcium homeostasis and bone turnover, has anti-proliferative, pro-differentiation, anti-bacterial, immunomodulatory and anti-inflammatory properties in various cells and tissues.

The D hormone [1α,25(OH)2D], regulated in endocrine, autocrine and paracrine manner, must be bound to the specific nuclear vitamin D receptor (VDR) to exert epigenetic and genetic effects influencing more than 2000 genes in all tissues and immune cells, essential for proliferation, differentiation and immunoregulation. VDR agonists inhibit in myeloid DCs, but not in plazmacytoid DCs, expression of surface co-stimulatory molecules such as MHC class II, CD40, CD80 and CD86. In T cells, 1,25(OH)2D decreases the production of IL-2, IL-17 and interferon-γ (IFNγ) and attenuates the cytotoxic activity and proliferation of CD4+ and CD8+ T cells, and promote the development of FoxP3+ regulatory T (TReg) cells and IL-10-producing T regulatory type 1 cells. In contrast to glucocorticoids which are non-selective immunosuppressive compaunds, 1α,25(OH)2D induces monocyte proliferation and the expression of interleukin-1 (IL-1) and cathelicidin (an antimicrobial peptide) by macrophages, thereby contributing to innate immune responses to some bacteria. Additionally it was shown that 1α,25(OH)2D can override steroid resistance and antagonize its negative bone turnover influence.

The discovery of the immunomodulatory and anti-tumor properties of D-hormone prompted researchers to investigate possibility of its use as a preventive and therapeutic agent for different autoimmune and malignant diseases. Several publications in last years have found a high prevalence of vitamin D deficiency in JIA, SLE and other chronic inflammatory diseases in childhood, with the mean values of 25(OH)D levels at the lower end of ‘acceptable’ range. Measurement of 25(OH)D level (as the only standardized toll to estimate vitamin D status) is actually only the reflection of the balance between food and/or supplement vitamin D diet intake and its utilization in the local tissues into the active D hormone (especially immune cells in the state of chronic inflammation). Recent recommendations and clinical guidelines have suggested vitamin D supplementation of up to 2000 IU/d to be safe and well tolerated in children with chronic diseases.

New recommendations and guidelines for vitamin D supplementation, as adjunct treatment option in JIA and other chronic inflammatory rheumatic diseases, is an appealing need due to its pleiotropic effects. It can both minimize bone fragility and contribute improvement of impaired immunity regulation in rheumatic diseases in children, especially those with necessity for long term steroid treatment.

## Disclosure of interest

None declared.

